# Enlargement of the Lymphatic Glands

**Published:** 1868

**Authors:** 


### ENLARGEMENT OF ALL THE LYMPHATIC GLANDS.—(Hodgkin's Disease.)

At page 383 of the present number will be found an interesting case of this, reported by Dr. John J. Black. We find another recorded by Dr. Wm. Carson, in our esteemed cotemporary, the *Western Journal of Medicine*, (Feb. 1868) and which we quote as an interesting contribution to our knowledge of this affection.

The subject of it was a woman twenty-eight years of age; unmarried, very pale, thin, and feeble, and having the physiognomy of protracted illness. Limbs wasted, breasts hard and disproportionately full, abdomen prominent and tolerably tense. Superficial, uneven and nodulated enlargements or tumors, hard and painless, were to be felt everywhere over the surface of the abdomen, and were evidently enlarged lymphatics. They were more numerous above the pubis, between the breasts, and towards the axillæ. The mammary glands were also affected by the same deposit.—Each nodule was readily defined, the skin was movable over it, and not discolored. During her early stay in the hospital, and before the effusion into the abdomen became too large, uneven and hard masses were to be felt within the abdominal cavity. They were larger than those on the surface, and grew rapidly. The glands of the neck were also enlarged, but not to the same extent as in the other regions.

The history that she gave of herself was, that she had enjoyed ordinary health until about two months since, when she noticed some changes in her breasts, and the beginning of the small, tumour-like swellings over the lower chest and abdomen. Pains in the abdomen were occasionally felt, and the abdomen began to enlarge, both on account of the irregular and hard growths and on account of some effusion.—Her strength began to fail rapidly, and she had been confined to her bed two weeks before her admission into the hospital.

"The treatment was determined by the two most obvious facts in the case: first, the general cachectic condition and the progressing disease in the abdomen, or rather the secondary effects of it, in producing dropsy. The prognosis was certainly fatal, and probably within a short period.—Tonics of various kinds and diuretics were used without effect. Evidences of the implication of the thoracic organs began to appear, in cough with mucous expectoration, and some dulness in the percussion sound of the chest, in limited portions both anteriorly and posteriorly. There was no acute pain, and no great disturbance of the respiratory act. The heart acted feebly but regularly, and without abnormal sound.

"She rapidly sank within three weeks from the time of her entrance into the hospital. The duration of her case, counting from her date of the beginning of her troubles, was about ten or twelve weeks.

"The *post mortem* examination showed, beginning with he thorax—externally as we have before said—nodulated masses on the surface between the breasts, in the mamæ and in the axillæ, each distinct, and of sizes from an almond to a walnut, with the skin movable over them. The lymphatics of the neck were enlarged. In the cavity of the thorax there were nodules scattered over the costal and pulmonary pleura, of varying size, some quite large. In the parenchyma of the lung were the same, with more tendency to infiltration. The pericardium also had a few. There was slight effusion into the pleural cavities. The heart was healthy. In the abnominal cavity not a single organ escaped. The liver was particularly affected, and contained numerous irregular masses of the same hard, cartilaginous substance, both on its surfaces and in its substance. The spleen was less affected than the liver, and the stomach still less. In the lumbar regions the masses were enormous.—The uterus could scarely be distinguished, and the pelvis was filled with the masses. The mesenteric glands were much enlarged, and the mesentery was almost a solid mass.

"The morbid growths cut with a hard, gristly feel, with rather a dry surface, and had the physical and microscopical appearance of scirrhus. We regret to say we did not make a thorough examination of the enlarged lymphatics."—*Am. Journal.*

